# Clinical Facts Relative to the Administration of Cod Liver Oil

**Published:** 1851-06

**Authors:** 


*Clinical facts relative to the administration of Cod Liver Oil.* By William H. Anderson, M. D., of Mobile.—So much has been written about this remedial agent, during the last few years, and so many exaggerated reports of its efficacy have been put before the public, that it is with some delicacy that I approach it, as a subject for further investigation. Nevertheless, medical *facts* are always valuable, and the usefulness of the profession depends mainly on their publicity and extensive circulation. At the present moment, there seems to be a division of opinion among medical men, as to the remedial power of Cod Liver Oil in any disease. Very respectable authority might be quoted, for its being inert in disease, and for its possessing no other qualities than other nutritive oils. The mass of medical testimony, however, is decidedly in its favor, and would seem to accord to it quite an elevated position in the pharmacopœia.

At this age, when physical diagnosis has attained so high a degree of perfection, it is indeed strange, why there should be such discrepancy about the reports of Cod Liver Oil, as a remedial agent in phthisis. If percussion, and particularly auscultation, reveal a certain extent of disease in any given portion of the lung, before using the remedy in question, and this same portion be carefully examined after using it, it certainly would seem that physical diagnosis was perfect enough to appreciate the change, if any had taken place; and yet, practitioners who stand high in the science, publish very discrepant reports on the subject. This is very much to be regretted, because the large mass of practitioners have not the opportunity to experiment largely for themselves, and they are compelled to regulate their practice by the experience of those who enjoy superior advantages. Discrepancy, therefore, among this latter class, on subjects which ought to be reduced to absolute demonstration, seriously injures our medical character, and detracts from the value of our professional periodicals.

The following summary of a few facts relative to the remedy which heads this article, may be relied on as authentic, the author having been placed under circumstances which enabled him to have a good deal of clinical experience with Cod Liver Oil.

The most important disease, for the alleviation and cure of which the oil has been used, is phthisis. Of 127 cases of this disease, which I particularly noticed, no other remedy was used. Of this number, the respective condition of the patients was as follows:

Sixty cases were in the incipient stage — prolonged respiration, slight cough, very little expectoration, no laryngeal disease except in five instances, dullness under one or both clavicles in every case, spitting of blood to a small extent in 18 cases — to a much greater in 4; organic disease of the heart in 9 of the whole number; functional disease in 12; organic disease of the liver, (supposed to be fatty degeneration,) in 4 patients; impaired digestion in 19; good digestion in the rest; evident emaciation in 20; considerable embonpoint in the other 40; two-thirds of the 60 were of the sanguine and lymphatic temperament — the other third were of the bilious and nervous, generally mixed. As nearly as could be traced, 41 were descendants of phthisical parents, principally, however, on the maternal side; 38 had been temperate, and well nourished from infancy, while the other 22 had lived a life of wretchedness, exposure, and intemperance — badly fed, badly clothed, and without any regular homes.

The 67 cases remaining to be accounted for, were far beyond the incipient stage of the disease. All had cavities in the lungs, of greater or less dimensions; 15 had small cavities with diffused dullness at the apex of either one lung or the other; 22 gave auscultatory signs of very large excavations, accompanied by functional or organic disease of the heart — colliquative diarrhœa, distressing cough, enlargement of the bronchial glands, chronic laryngitis, great prostration and emaciation. Of these 22, 14 had enlarged livers, and 5 tubercular degeneresence of the mesenteric glands; 15 of them were females under the age of 30. The whole number were adults.

The remaining cases were in the last stage of the disease, and all of them died not long after admission. Exclusive of the deaths, therefore, there were 97 cases of phthisis treated salely by cod liver oil for the period of three months. Out of the 60 mentioned as being in the incipient stage, there was an evident amelioration in 44, but in no single instance did the dullness leave the affected region. The cough and expectoration were alleviated, and the majority of those affected with functional disease of the heart were relieved of this symptom. The amelioration was most evident in those patients who were of the sanguino-lymphatic temperament. We cannot say, then, that any one patient was cured, but that the disease was arrested, and that the general condition was improved, there is no doubt. How long the arrest lasted, it was impossible to tell, but it was the opinion of the highest medical authority of France, that many of this number might be permanently relieved by change of climate, occupation, &c. The prolonged expiration was closely noticed, and it was thought that in some instances it was removed, in others, lessened. The great delicacy, however, in properly appreciating this change, may have led the auscultators into error. Upon the whole, the exhibition of the remedy was very satisfactory both to the physician and the patient.

I have mentioned that 15 cases had small cavities with diffused dullness at the apex of either one or the other lung. Thirteen out of this fifteen improved under the treatment, and 9 improved enough to consider the disease, for the time, staved off. Of these, the cavities certainly did not increase, the general symptoms diminished, the patient gained flesh and strength, but the dullness on percussion remained. Two of the 15 seemed not to be affected by the oil, indeed, but both of them had a sort of intolerance of it, and took it only in small quantities. The greatest quantity taken by any one patient amounted to three-quarters of a pint per day. The average dose was about one ounce, three times a day.

Of the 22 patients who had large cavities, colliquative diarrhœa, dullness, cough, &c. there was manifest improvement noticed in 11. This improvement consisted in increase of flesh and strength, in arrest of the diarrhœa, and great alleviation of the cough. The laryngitis, too, seemed much benefited. The other 11 were little affected by the treatment, although the remedy had a fair trial. After three months, the chance of death or recovery amounted to about the same it did when they entered. The immediate fatality of the disease, however, on the first 11, was undoubtedly postponed — to what period of time, could not be ascertained."

The author gives results of the use of the oil in several other affections. From transactions of Alabama State Medical Association.

Ed. Buff. Jour.

